# Optogenetic Control of PIP2 Interactions Shaping ENaC Activity

**DOI:** 10.3390/ijms23073884

**Published:** 2022-03-31

**Authors:** Tarek Mohamed Abd El-Aziz, Amanpreet Kaur, Mark S. Shapiro, James D. Stockand, Crystal R. Archer

**Affiliations:** 1 Department of Cellular and Integrative Physiology, University of Texas Health Science Center at San Antonio, San Antonio, TX 78228, USA; mohamedt1@uthscsa.edu (T.M.A.E.-A.); shapirom@uthscsa.edu (M.S.S.); stockand@uthscsa.edu (J.D.S.); 2 Faculty of Science, Zoology Department, Minia University, El-Minia 61519, Egypt; 3 Department of Chemistry, University of Washington, Seattle, WA 98195, USA; amanpreet.kaur1223@gmail.com

**Keywords:** ENaC, phosphoinositides, PIP2, optogenetic, CRY2, sodium channel, CoroNa Green

## Abstract

The activity of the epithelial Na^+^ Channel (ENaC) is strongly dependent on the membrane phospholipid phosphatidylinositol 4,5-bisphosphate (PIP2). PIP2 binds two distinct cationic clusters within the N termini of β- and γ-ENaC subunits (βN1 and γN2). The affinities of these sites were previously determined using short synthetic peptides, yet their role in sensitizing ENaC to changes in PIP2 levels in the cellular system is not well established. We addressed this question by comparing the effects of PIP2 depletion and recovery on ENaC channel activity and intracellular Na^+^ levels [Na^+^]_i_. We tested effects on ENaC activity with mutations to the PIP2 binding sites using the optogenetic system CIBN/CRY2-OCRL to selectively deplete PIP2. We monitored changes of [Na^+^]_i_ by measuring the fluorescent Na^+^ indicator, CoroNa Green AM, and changes in channel activity by performing patch clamp electrophysiology. Whole cell patch clamp measurements showed a complete lack of response to PIP2 depletion and recovery in ENaC with mutations to βN1 or γN2 or both sites, compared to wild type ENaC. Whereas mutant βN1 also had no change in CoroNa Green fluorescence in response to PIP2 depletion, γN2 did have reduced [Na^+^]_i_, which was explained by having shorter CoroNa Green uptake and half-life. These results suggest that CoroNa Green measurements should be interpreted with caution. Importantly, the electrophysiology results show that the βN1 and γN2 sites on ENaC are each necessary to permit maximal ENaC activity in the presence of PIP2.

## 1. Introduction

**Figure 1:**
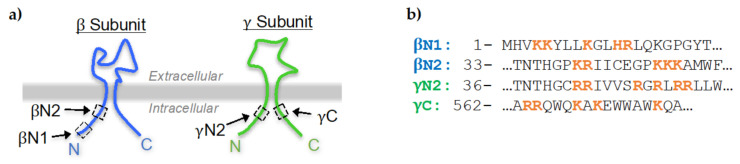
Cationic sites in ENaC tested for PIP2 binding. (**a**) Schematic of the ENaC subunits with the sites tested for PIP2 binding, βN1, γN2, γC, and βN2, indicated by dashed boxes. Adapted from Archer et al., 2020 [13]. (**b**) Human ENaC sequences corresponding to βN1, γN2, γC, and βN2. The cationic residues neutralized to alanine are indicated in boldface orange font.

The epithelial Na^+^ channel, ENaC, is a trimeric channel that closely resembles the chalice-like structure of the closely related acid sensing ion channel (ASIC) [1,2]. ENaC is comprised of 3 independent proteins subunits, called α, β, and γ, which are encoded by three distinct genes [3,4,5]. ENaC conducts Na^+^ across tight epithelia such as those lining the lungs and kidney tubules [3]. ENaC is necessary for liquid clearance in the lungs and consequently, the knockout of α-ENaC in mice is lethal. In contrast, the overexpression of β-ENaC follows a pattern of cystic fibrosis [6]. In the kidney, ENaC is the final arbiter of Na^+^ reabsorption. Pathological disturbance of ENaC results in blood pressure disorders such as Liddle’s syndrome [7,8]. The body’s dependence on Na^+^ homeostasis underscores the importance of the proper function of the mechanisms that regulate ENaC. Many of these mechanisms regulate ENaC by acting on its intracellular domains. Each ENaC subunit resembles a hairpin structure with a large extracellular globular domain anchored by two transmembrane domains [1]. Each transmembrane domain connects to relatively short intracellular N and C terminal tails comprising 55–85 amino acids; thus, ENaC has 6 intracellular domains. Although the bulk of ENaC has been elucidated using cryo-electron microscopy, the structure of the intracellular domains remains obscure [1]. Structure prediction software and experimental observations indicate that these intracellular domains are likely largely disordered yet they may adopt helical structure when interacting with other regulatory cofactors, such as the membrane phospholipid, phosphatidylinositol 4,5-bisphosphate (PIP2) [9]. PIP2 is a low abundance phospholipid in the plasma membrane that is necessary for ENaC to be maximally open [10,11,12]. Earlier studies showed that mutation of polybasic clusters in ENaC subunits reduces its ability to respond to changes in PIP2 [11]. More recently, we showed that PIP2 binds two of these distinct clusters located on the intracellular termini of β and γ subunits of ENaC, referred to here as the βN1 and γN2 sites [13]. PIP2 consists of a phosphorylated, cytoplasmic inositol ring (called the PIP2 headgroup) which is anchored to the plasma membrane via phosphodiester linkage to two fatty acyl chains embedded within the inner leaflet of the membrane [14]. The PIP2 headgroup bears two phosphoryl groups covalently bound to its carbons at positions 4 and 5 (C4 and C5) [14,15,16]. Those phosphoryl groups can be reversibly removed or added by site-specific lipid phosphatases and kinases [17]. This study takes advantage of the ability of the phosphatase OCRL to deplete PIP2 by removing the C5 phosphoryl group [18]. The anionic PIP2 headgroup forms electrostatic interactions with cationic amino acid residues of proteins [14] and is hypothesized to bind the βN1 and γN2 sites on ENaC. βN1 is located on the extreme, intracellular N terminus of β-ENaC (Figure 1a, left). γN2 is located on the intracellular N terminus of γ-ENaC, near the inner plasma membrane (Figure 1a, right). In our previous work, we reported that the equilibrium constant of PIP2 for βN1 is K_d_ ~5 μM, and for γN2 is K_d_ ~13 μM [13]. The γC site of γ-ENaC (Figure 1a, right) is a potential PIP2 interaction site because it demonstrated an interesting profile in binding experiments but had very weak affinity for PIP2 [13]. 

In this study, we investigated how mutations made to βN1, γN2 and γC affect the activity of the human ENaC transfected in mammalian cells. We mutagenized the channel by substituting alanine for each cationic residue within these domains, indicated in bold orange letters in Figure 1b. We used the optogenetic dimerization pair CIBN-CAAX and mCherry-CRY2-OCRL (mCh-CRY2-OCRL), which has been previously used to examine the effects of PIP2 depletion on ion channel activity [13,18]. CIBN-CAAX is localized to the plasma membrane via the CAAX moiety. mCh-CRY2-OCRL is rapidly recruited from the cytoplasm to the plasma membrane by dimerization with CIBN, in response to blue light illumination (BLI) [18]. The 5-phosphatase OCRL is a protein that selectively removes the carbon 5 phosphoryl group of PIP2 [17,19]. Membrane-localized mCh-CRY2-OCRL rapidly depletes PIP2. mCh-CRY2-OCRL reversibly translocates to the cytoplasm in the absence of blue light within ~5–7 min, after which PIP2 levels are immediately replenished [20,21,22]. We previously used the CIBN/mCh-CRY2-OCRL system in HEK 293 cells to show that BLI-induced PIP2 depletion caused a reduction of intracellular Na^+^ levels ([Na^+^]_i_) in cells expressing wild type ENaC (wtENaC) [13]. Here, we used the CIBN/mCh-CRY2-OCRL system to show that the βN1 and γN2 sites play a strong role in sensitizing ENaC to changes in PIP2 levels in the cellular system. Channel activity was measured by two different methods. First, we used the Na^+^ selective fluorescent indicator, CoroNa Green AM, to track changes in [Na^+^]_i_ of cells expressing mutant ENaC, in response to BLI. We compared those results to the direct measurements of mutant ENaC activity in response to BLI using whole cell patch clamp electrophysiology. The purpose of this study was to determine whether each independent PIP2-ENaC interaction site is sufficient to abolish the response of ENaC to changes in PIP2 levels, and to evaluate CoroNa Green as a tool for studying Na^+^ ion channel activity.

## 2. Results

### 2.1. Mutations of the PIP2-ENaC Binding Sites Do Not Affect Membrane Expression of ENaC

We measured the membrane expression of ENaC using Total Internal Reflection Fluorescence (TIRF) microscopy. In TIRF, the excitation light is nearly totally and internally reflected at the interface between two media transparent to light with different refractive indices, in this case glass (~1.52) and aqueous solution (1.33). However, an “evanescent wave” does propagate into the second medium normal to the plane of the interface, but decays exponentially over a short distance with a length constant of ~300 nm, thus allowing only fluorescent proteins located within that distance from cells adherant to a glass coverslip to be excited [23,24]. We used TIRF microscopy to determine if the mutations used in this study (Figure 1b) affected the membrane expression of α-, β- and γ-ENaC subunits. HEK 293 cells were transiently transfected with cDNA encoding ECFP-tagged α-ENaC (CFP-αENaC), along with EYFP-tagged β-ENaC (βENaC-YFP) and mCherry-tagged γ-ENaC (γENaC-mCherry) constructs containing wild-type sequences or mutations to the indicated cationic clusters. The data shown in Supplemental Figure S1a,b show that all three subunits were expressed at the plasma membrane in the same cell. Cells expressing the γN2 mutants had reduced membrane expression of γ-ENaC, whereas α- and β-ENaC expression was similar regardless of whether it was part of the wt or mutant channel. Moreover, the membrane expression and activity of ENaC was unaffected by the expression of mCh-CRY2-OCRL under dark (normal PIP2) conditions (Supplemental Figure S1c,d).

### 2.2. PIP2 Depletion Reduces [Na^+^]_i_ and Current Density in Cells with wtENaC

**Figure 2:**
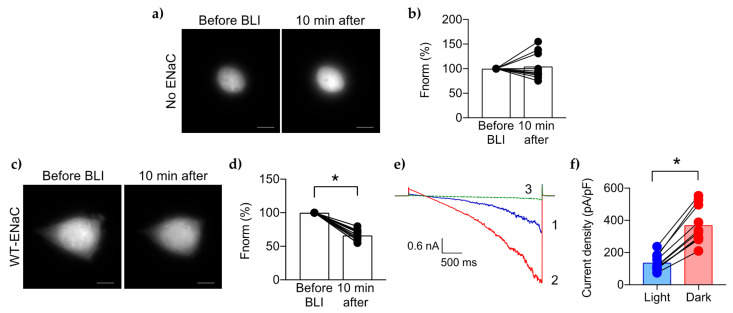
PIP2 depletion decreases [Na^+^]_i_ and current density of wtENaC. Representative micrographs show the CoroNa Green uptake in HEK 293 cells without transfection (**a**) or transfected with wtENaC (**c**). Summary graphs of changes in the mean [Na^+^]_i_ of cells with no ENaC (n = 11 cells) (**b**) or wtENaC (n = 11) (**d**), as measured by CoroNa intensity excited at 514 nm, 10 min after BLI, normalized to CoroNa levels before BLI. (**e**) Representative current trace showing wtENaC activity under BLI (1, blue trace), followed by Dark (2, red trace), and finally with application of 10 µM amiloride (3, green trace). (**f**) Summary graph of the mean current density of wtENaC at −100 mV under low PIP2 levels (Light, blue bar and blue circles) vs. maximum PIP2 levels after 10 min (Dark, red bar and blue circles) (n = 10). For each graph, the line drawn between 2 circles represents an independent cell under different PIP2 levels. * Indicates *p* < 0.0001 determined by the paired *t* test.

To quantify changes of [Na^+^]_i_, we incubated cells with the fluorescent cell-permeable Na^+^ indicator, CoroNa Green AM, where high intensity of emission corresponds to an increase of [Na^+^]_i_ and decreased intensity corresponds to decreased [Na^+^]_i_. CoroNa Green is excited between 440–514 nm with peak emission at 490 nm [25]. CIBN-CRY2 dimerization occurs between 405 and 500 nm [20]. Therefore, we used brief laser excitation at 514 nm to excite CoroNa Green with minimal impact on PIP2 levels [22]. TIRF microscopy was used to capture these intracellular changes in local [Na^+^]_i_ which are expected to occur just inside the membrane near ENaC while changes in global [Na^+^]_i_ would not be easily detected. HEK cells expressing CFP-αENaC (excited with a 445 nm laser), untagged β-ENaC, untagged γ-ENaC, untagged CIBN-CAAX and mCh-CRY2-OCRL (excited with a 561 nm laser) were used for the ENaC-PIP2-response experiments. The cells were then pulsed with blue light (BLI, 445 nm laser) to stimulate mCh-CRY2-OCRL translocation to the membrane and subsequent PIP2 depletion. The presence of CFP-αENaC was confirmed in each cell during the BLI step. The representative micrographs and summary graph of CoroNa Green intensity in Figure 2a,b show that there was no change in the mean [Na^+^]_i_ in control cells with no ENaC, in response to BLI. In contrast, BLI-induced depletion of PIP2 caused a significant reduction of [Na^+^]_i_ (~34% decrease) in cells expressing wtENaC, indicating that ENaC is strongly dependent on PIP2 (Figure 2c,d). These results are consistent with our previous findings that the ENaC-specific channel blocker amiloride blocked the reduction of [Na^+^]_i_ caused by BLI-induced depletion of PIP2 in cells expressing wtENaC [13].

**Figure 3:**
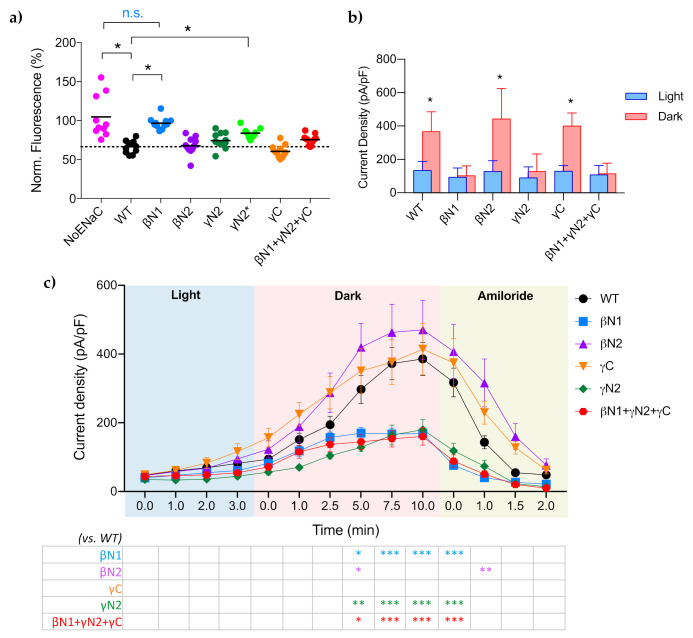
Summary of mutant-ENaC responses to PIP2 depletion and recovery. (**a**) Summary graph comparing the CoroNa Green intensity of cells expressing each mutant 10 min after BLI. *, *p* ≤ 0.0001, n.s., no significant difference. The dashed line indicates the mean CoroNa Green intensity of wtENaC 10 min after BLI. (**b**) Summary graph comparing the mean current densities ± SD of each ENaC under BLI-induced PIP2 depletion (Light/blue) and PIP2 recovered (Dark/red). *, *p* < 0.0001, comparing light to dark. (**c**) Summary timeline of the current density of each mutant ENaC in response to recovery of PIP2 levels after CRY2-OCRL relocation to the cytoplasm, expressed as the mean of 5–7 cells ± SEM. Two-way ANOVA (column factor, mutant; row factor, time) indicated significant differences between 5 min dark and 1 min after amiloride, where ***, *p* < 0.0001, **, *p* < 0.005, and *, *p* < 0.05, compared to wtENaC, noted in the table below the time plot.

For patch clamp experiments, we used Chinese hamster ovary (CHO) cells because they have no detectable background ion channel activity. The cells were grown on glass chips coated with poly-l-lysine (PLL). Since PLL has been used previously as a PIP2 scavenger in excised patches, we compared the activity of wtENaC on glass chips coated with PLL to glass chips coated with gelatin. Supplemental Figure S2 shows that the PIP2-dependent activity of ENaC in cells grown on PLL was similar to those grown on gelatin. We started patch clamp recordings in the presence of transmitted light and BLI-induced PIP2 depletion in order to find the cells expressing both mCherry and ECFP and to visually guide the patch pipette to the cell surface. BLI was performed using an epifluorescence microscope equipped with a mercury lamp and standard ECFP and mCherry filter cubes. Macroscopic ENaC currents were recorded during voltage ramp from 0 to −100 mV. The current density at −100 mV after 10 minutes was reported. Under this protocol, wtENaC exhibited low current density under BLI (~137 ± 50 pA/pF) after 10 min, shown in Figure 2e, blue trace; Figure 2f, blue bar. After 3 min of recording under BLI, we turned off the lights to both the microscope and the room, to allow translocation of mCh-CRY2-OCRL to the cytoplasm followed by recovery of PIP2. The activity of wtENaC immediately began to increase, reaching a peak current density of 371 ± 117 pA/pF at 10 min (Figure 2e, red trace; Figure 2f, red bar). Figure 2f summarizes the voltage ramp recordings taken at 10 min under depleted PIP2 (blue bar) and recovered PIP2 level (red bar). The time course of this PIP2-dependent ENaC recovery is described in further detail in Figure 3c, and later in Section 2.5. Perfusion with 10 μM of the selective ENaC blocker, amiloride, abolished wtENaC activity demonstrating that the PIP2-dependent currents are solely from ENaC (Figure 2e, green trace). The peak current density of wtENaC under recovered PIP2 was similar to wtENaC under normal conditions without mCh-CRY2-OCRL (Supplemental Figure S3). This result shows that the CIBN/mCh-CRY2-OCRL system is effective for measuring ENaC response to PIP2 levels. These results further indicate that the presence of cytoplasmic CRY2-OCRL does not affect normal PIP2 levels and PIP2 is replenished to its normal levels in the dark.

### 2.3. Cellular-Mutagenesis Validates the βN1 Site as a PIP2 Binding Site

**Figure 4:**
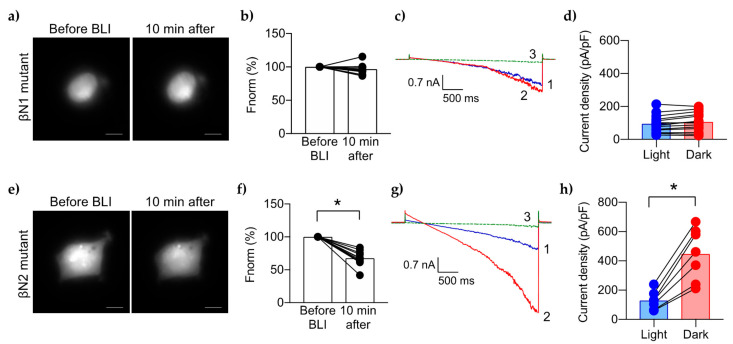
Effects of PIP2 depletion on mutant β-ENaC. Representative micrographs showing the CoroNa Green uptake in HEK 293 cells transfected with mutant βN1-ENaC (**a**) or mutant βN2-ENaC (**e**). The graphs in (**b**,**f**) summarize changes in the mean [Na^+^]_i_ as determined by CoroNa Green fluorescence levels (n = 11 per group). Representative current traces of the βN1 mutant (**c**), or the βN2 mutant (**g**), under BLI (1, blue trace), then dark (2, red trace), then 10 µM amiloride (3, green trace). Summary graphs of the mean current density for the βN1 mutant (n = 14) (**d**), or the βN2 mutant (n = 7) (**h**), at −100 mV under low PIP2 levels (“Light”, blue bar and blue circles) vs. maximum PIP2 levels (“Dark”, red bar and red circles). For each graph, the line drawn between 2 circles represents an independent cell under different PIP2 levels. *, *p* < 0.0001 determined by paired *t* test.

We previously reported that a peptide corresponding to the βN1 site of ENaC has moderate affinity for PIP2 (K_d_ ~5 μM) [13]. To determine if βN1 is critical for ENaC activity in a PIP2-dependent manner, we tested the mutant βN1-ENaC in the optogenetic cellular assay. After BLI, CoroNa Green emission remained unchanged 10 min after BLI-induced PIP2 depletion (Figure 4a,b). For whole cell patch clamp electrophysiology, the mean current density of mutant βN1-ENaC was similar to wtENaC under BLI-induced PIP2 depletion (95 ± 53, Figure 4d, vs. 137 ± 50 pA/pF, Figure 2f blue bars). Switching to dark conditions allowed for full PIP2 recovery, yet the current density of mutant βN1-ENaC only slightly increased to 106 ± 57 pA/pF up to 10 min in the dark (Figure 4c,d, red; Figure 3a,c, blue circles). Mutant βN1-ENaC exhibited the same basal activity with normal PIP2 levels in the absence of CIBN/mCh-CRY2-OCRL (Supplemental Figure S3). In both of these experiments, mutant βN1-ENaC did not respond to the controlled changes in PIP2 levels, suggesting that the βN1 site is necessary for the PIP2-ENaC interactions. 

Mutations were also made to the βN2 site of ENaC. Although this site also contains several cationic residues, the peptide corresponding to this domain did not directly bind PIP2 in our earlier study, and was used here as a control [13]. Figure 4e,f shows that the CoroNa Green intensity decreased by ~32%, similar to the wtENaC response. The current traces also followed the same pattern as wtENaC, with very low activity during BLI-induced PIP2 depletion followed by a large increase in channel activity after PIP2 recovery in the dark (Figure 4g). The summary graph in Figure 4h shows the mean current density under PIP2 depletion was 129 ± 63 pA/pF and increased to 447 ± 180 pF/pA under PIP2 recovery, which is similar to wtENaC activity. We also observed high ENaC activity in cells without CIBN/mCh-CRY2-OCRL (Supplement Figure S3). These results are consistent with the binding data showing βN2 site is not a PIP2 binding site. 

### 2.4. Cellular-Mutagenesis Assays Confirm That γN2, but Not γC, Binds PIP2

**Figure 5:**
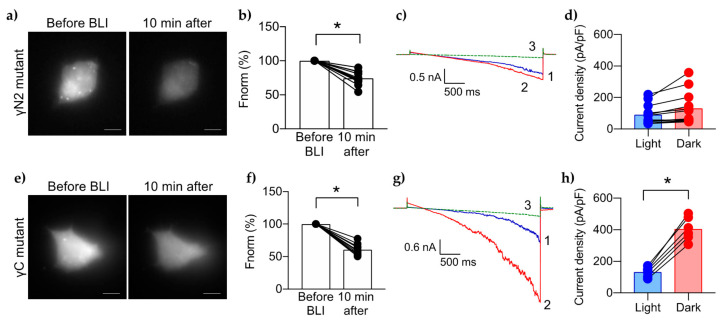
Effects of PIP2 depletion on mutant γ-ENaC. Representative micrographs showing the CoroNa Green fluorescence levels in HEK 293 cells transfected with ENaC containing alanine substitutions to the γN2 site (**a**) or the γC site (**e**). Summary graphs (**b**,**f**) of changes in the mean [Na^+^]_i_ as determined by CoroNa Green fluorescence levels. Representative current traces of the γN2 mutant (**c**) or the γC mutant (**g**) under BLI (1, blue trace), then dark (2, red trace), then 10 µM amiloride (3, green trace). Summary graphs of the mean current density for the γN2 mutant (**d**) or the γC mutant (**h**) at −100 mV under low PIP2 levels (“Light”, blue bar and blue circles) vs. maximum PIP2 levels (“Dark”, red bar and red circles). *, *p* < 0.0001 determined by paired *t* test.

The γN2 peptide also has a moderate biochemical affinity for PIP2 (K_d_ ~13 μM) [13]. Therefore, we expected the mutant γN2-ENaC would produce results similar to the mutant βN1-ENaC. Surprisingly, BLI-induced PIP2 depletion caused a ~26% decrease of CoroNa Green intensity in cells expressing mutant γN2-ENaC (Figure 5a,b). The mean CoroNa Green intensity of mutant γN2-ENaC after 10 min was similar to wt ENaC (Figure 3a). However, the mutant γN2-ENaC had low current density under BLI-induced PIP2 depletion, which was similar to mutant βN1-ENaC (Figure 5c, blue trace and Figure 5d, blue bar). The current density did not significantly increase after full PIP2 recovery in the dark (92 ± 63 pA/pF vs. 131 ± 102 pA/pF, respectively) (Figure 5c, red trace and Figure 5d, red bar). The activity of mutant γN2-ENaC also remained low in cells without CIBN/mCh-CRY2-OCRL (Supplemental Figure S3). 

To better understand why cells expressing the mutant γN2-ENaC had a strong decrease of CoroNa Green but no change in current density in response to changes in PIP2, we examined the CoroNa Green emission under normal PIP2 levels. As shown in Supplemental Figure S4, the CoroNa Green emission of mutant γN2 cells was low CoroNa Green at the beginning of each experiment compared to wtENaC (1024 ± 1044 vs. 9197 ± 1267 AU) (Figure S4a). Nether had a significant difference in CoroNa Green emission after 10 min. Moreover, CoroNa Green emission of mutant γN2-ENaC cells were ~83% lower than cells expressing wtENaC or mutant βN1-ENaC (Figure S4b). These data suggest that CoroNa Green had less uptake in cells expressing mutant γN2-ENaC and that the Na^+^ indicator may have leaked from those cells a faster rate than the experimental time. This reduced CoroNa Green emission could explain why cells expressing γN2-ENaC do not correspond with patch clamp data. Since the starting values of the Na^+^ indicator were so low compared to the other cells tested, we hesitate to strongly rely on the CoroNa data for this mutant.

We also examined the CoroNa Green response in a partial mutant, γN2*, which contained the substitutions R48A/R50A/R52A/R53A of human γ-ENaC, in contrast to the γN2 mutant that included the substitutions R42A and R43A. We found that cells expressing the γN2* mutant had a smaller decrease of CoroNa Green intensity in response to BLI (16 ± 7%) after BLI-induced PIP2 depletion, which was also significantly less change of intensity than wtENaC (Supplemental Figure S5 and Figure 3a). Cells expressing mutant γN2* had normal starting levels of CoroNa Green (Supplemental Figure S4b), but the mutant γN2* subunit also had reduced membrane expression (Supplemental Figure S1). These results suggests that PIP2 interacts with the “…*SRGRLRRL*…” sequence of the γN2 site, which is closer to the transmembrane domain TM1 of γ-ENaC. Together, these results indicate the γN2 site is critical for PIP2 regulation of ENaC.

The γC domain of ENaC was previously reported to be a PIP2-binding candidate. Its weak affinity (K_d_ ~800 μM) suggested that it might play a moderate to small role in the ENaC response to changes in PIP2 levels. However, we found that PIP2 depletion caused a significant decrease of CoroNa Green intensity ~40 ± 8% in cells expressing mutant γC-ENaC (Figure 5e,f). This decrease was similar to wtENaC. In addition, the current density of mutant γC-ENaC fully recovered to 406 ± 75 pA/pF within 10 min after PIP2 recovery in the dark (Figure 3c and Figure 5g,h). These results are consistent with an earlier study from our lab showing that the current density from mutant mouse γC-ENaC is similar to wtENaC [11]. These results strongly suggest that the γC domain is not involved in PIP2 binding or regulation of ENaC. 

### 2.5. Mutation of All 3 PIP2 Binding Candidates Does Not Further Increase PIP2 Sensitivity

**Figure 6:**
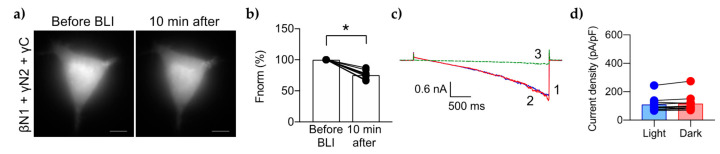
Effects of PIP2 depletion on the triple mutant. Representative micrographs showing the CoroNa Green fluorescence levels in HEK 293 cells transfected with triple mutant ENaC containing alanine substitutions to the βN1, γN2, and γC sites (**a**). Summary graph (**b**) of changes in the mean [Na^+^]_i_ as determined by CoroNa Green fluorescence levels. Representative current traces of the triple mutant (**c**) under BLI (1, blue trace), then dark (2, red trace), then 10 µM amiloride (3, green trace). Summary graph of the mean current density for the triple mutant (**d**) at −100 mV under low PIP2 levels (“Light”, blue bar and blue circles) vs. maximum PIP2 levels (“Dark”, red bar and red circles). *, *p* < 0.0001 determined by paired *t* test.

We mutagenized the three candidate PIP2 sites on ENaC to create the triple mutant, βN1-γN2γC-ENaC. Since cells expressing mutant βN1-ENaC had no change of CoroNa Green intensity in response to BLI-induced PIP2 depletion, we expected to see similar results with this triple mutant. Instead, the CoroNa Green intensity of cells expressing the triple mutant were reduced by 25% ± 7% (Figure 6a,b). Cells expressing the triple mutant had low starting levels of CoroNa in contrast to the other cells, but similar to mutant γN2-ENaC (Supplemental Figure S4b). In patch clamp experiments, the triple mutant exhibited low mean current density (111 ± 52 pA/pF) under BLI-induced PIP2 depletion, with no change in response to PIP2 recovery in the dark (118 ± 60 pA/pF) (Figure 6c,d). The activity of this triple mutant was also low in cells without CIBN/mCh-CRY2-OCRL (Supplemental Figure S3). This low basal activity of both wtENaC and mutant βN1-ENaC suggests that ENaC has a small, constitutive activity that is not dependent on PIP2. These results show that mutating βN1 and γN2 sites together results in a lack of ENaC response to PIP2 depletion that is not different compared to mutating the βN1 and γN2 sites on their own.

The graphs in Figure 3 summarize the effects of PIP2 depletion and recovery on the mutants tested in this study. Figure 3a shows that the Corona Green emission of cells expressing mutant βN1 were not different from the “No ENaC” control levels 10 min after BLI. Both βN1 and γN2* mutants had significantly less change of CoroNa Green than wtENaC. The mean change of CoroNa Green of cells expressing mutant γN2 and triple mutant were not found to be significantly different than wtENaC. The patch clamp results in Figure 3b revealed a clear distinction in the response of each mutant to changes in PIP2 levels. Each wt and mutant tested had very low residual activity ~100pA/pF under BLI-induced PIP2 depletion (blue bars), whereas wtENaC, mutant βN2-ENaC and mutant γC-ENaC displayed robust activity in response to PIP2 recovery in the dark (red bars). The maximal current density of mutant βN1- and γN2-ENaC and the triple mutant all remained low, with ~37% ENaC activity compared to wtENaC at normal PIP2 levels, after 10 min PIP2 recovery (Figure 3b,c—blue, green and red lines). This low activity under both normal (Supplemental Figure S3) and recovered PIP2 levels indicates that βN1- and γN2 are both necessary for PIP2 regulation of ENaC. 

In contrast, the PIP2-responsive ENaC constructs (wtENaC, and βN2 and γC mutants) had maximal activity after 7–10 min of recovery time in the dark (Figure 3c-black, purple and orange lines). The difference between the mutants at each time point, compared to wtENaC, are noted in the table below the chart. This timing is consistent with another study which reported a t_1/2_ = ~6.8 min for the mCh-CRY2-OCRL to dissociate from CIBN and redistribute back to the cytoplasm, followed by the resynthesis of PIP2 [18]. We also observed that all ENaC constructs in this study exhibited a minimal level of current density of ~100 pA/pF that was not dependent on PIP2 (Figure 3b and Supplemental Figure S3). These results indicate that the maximal current density of ENaC facilitated by PIP2 is dependent on the βN1- and γN2 sites; and ENaC produces a low basal level of activity that is not dependent on PIP2. This observation is in agreement with Pochynyuk et al, 2007 [13], which also reported that ENaC has basal activity that is independent of PIP2, and that PIP2 may serve a permissive role for increasing the open probability of ENaC.

## 3. Discussion

The results of this study validate our previous study showing that peptides corresponding to the βN1 and γN2 sites of ENaC bind to PIP2 [14]. The present study expands on this by investigating the effects of mutant βN1 and γN2 on the ENaC response to changes in PIP2 levels. Here, we show that mutating either βN1 or γN2 equally inhibit the ability of ENaC to reach maximal activity compared to wtENaC. The results suggest that PIP2 may interact with both βN1 and γN2 at the same time to permit ENaC maximal activity. Mutating either site alone prevents ENaC from responding to changes in PIP2 levels. We also observed a low residual ENaC activity of ~100 pA/pF in all mutants and wtENaC, even when PIP2 was depleted. This is consistent with earlier observations in our lab showing that PIP2 serves a permissive role for maximizing the P_o_ of ENaC [13]. These data indicate that PIP2 depletion would reduce ENaC activity rather than completely abolish its activity. 

Based on our results, CoroNa Green may be a useful method for testing Na^+^ ion channel activity or examining changes in [Na^+^]_i,_ but should be paired with patch clamp or other more rigorous methods. Careful attention should be made for designing control experiments to understand the efficiency of loading and retention of the Na^+^ indicator prior to starting experiments, and caution be made for interpreting the results. Indeed, mutation of the γN2 site of ENaC corresponded to reduced CoroNa Green emission, possibly due to less uptake into the cells or faster leakage from the cells, although it is unclear why (Supplemental Figure S4). 

Earlier studies suggest there may be interplay between PIP2 and ubiquitin in controlling ENaC. The βN1 site of ENaC carries several lysines that were previously reported to be targets of ubiquitination-induced protein turnover [26,27,28]. However, we observed no change of membrane ENaC levels with mutations to those lysines, compared to the membrane levels of wtENaC (Supplemental Figure S1). These data are consistent with a recent finding that ubiquitination of the β-ENaC subunit was minimal [29]. It was also previously reported that deletion of the βN1 site of mouse ENaC also had no change in membrane expression [11]. If mutating βN1 prevented the ubiquitination of ENaC, then we would expect increased expression at the membrane, however there was no change (Supplemental Figure S1). The results from the present study suggest that the βN1 site of ENaC may only be a PIP2 binding site, although it does not rule out the possibility of ubiquitin having other impact on ENaC activity involving the βN1, to include competition or interplay with PIP2.

ENaC has moderate biochemical affinity for PIP2 of K_d_ ~5 μM for βN1 and K_d_ ~13 μM for the γN2 site. Our data show that these moderate affinity levels are responsible for the high sensitivity of ENaC to PIP2 levels. A very high affinity would probably result in less response to PIP2. For example, Kv7.3 channels which have high apparent affinity and is less sensitive to depletion of PIP2 compared with Kv7.2 channels having lower apparent affinity and thus very sensitive to depletion of PIP2 [30,31]. It is still unclear whether both PIP2 sites form a single “binding pocket” by simultaneously binding PIP2 or if each site binds PIP2 independently of each other. Having insight to this question will further impact our understanding of how other signaling cofactors or genetic mutations modulate the PIP2-ENaC interactions. PIP2 regulates the activity of many K^+^ and Ca^2+^ ion channels, and many of these channels exhibit a defined PIP2-binding pocket comprised of distant cationic residues within their intracellular domains [16,32,33]. This makes a strong case for ENaC to also have a PIP2 binding pocket comprised of its βN1 and γN2 sites. We anticipate better clarification on this question as more structural studies emerge.

The γN2 site overlaps a nuclear localization sequence (NLS), that includes R42 and R43 within the human ENaC sequence “…HGCRR…”, shown in Figure 1b [34,35]. Our lab has reported that the N terminus of γ-ENaC becomes cleaved, then translocates to the nucleus or nucleolus [34]. While the cleavage site and function of free γN is unclear, an increased level of free γN leads to reduced macroscopic current density of ENaC. Mutating this NLS site appeared to play some role in CoroNa Green uptake and half life in the cells, given that cells expressing the γN2 mutant including R42A and R43A had reduced CoroNa Green uptake but cells expressing the γN2* mutant (without those mutations) had normal CoroNa Green uptake. These data could serve as the rationale for furthering the study of cleaved γN in regulating ENaC activity and how that might impact ENaC response to PIP2 depletion.

Acid sensing ion channels (ASICs) are within the same superfamily as ENaC [3,36]. A recent study of chicken ASIC1 reveals another interesting role for how PIP2 might facilitate Na^+^ permeation through ENaC [37]. As with ASIC1, each α-, β- and γ-ENaC subunit bears an "HG motif” in the N terminal “pre-TM1” domain adjacent to the N-terminal transmembrane domain (TM1) [38]. Genetic mutation of the HG motif (G37S) on β-ENaC results in pseudohypoaldosteronism I (PHA1B) by reducing the P_o_ of ENaC [39], underscoring the importance of the HG motif. In the homo-trimeric ASIC1, the preTM1 domains of each subunit loop back toward the TM1 domains in the plasma membrane and line the pore of the channel protein [37]. The histidines of the HG motifs lean towards the pore, constricting the lower permeation pathway in the desensitized and resting configurations of the channel. Interestingly, the PIP2 binding domain of γ-ENaC (γN2 ) is located between the HG motif and TM1 that closely align to this region of the ASIC sequence. If ENaC adopts the same pre-TM1 pore loop structure to allow its HG motifs to control the size of the lower permeation pathway, one could predict a direct role for PIP2 controlling ion permeation of ENaC by tugging this pre-TM1 loop to create an “open pore” configuration. We look forward to seeing future structural studies of ENaC shed more light on the interactions between PIP2 and ENaC.

## 4. Materials and Methods

### 4.1. Plasmids, Synthetic Peptides and Reagents

Plasmids containing mCherry-CRY2-OCRL and CIBN-CAAX were gifts from Pietro De Camilli (Addgene plasmid #66836 and #79574). Human ENaC (hENaC) constructs of untagged β and γ subunits in the pMT3 vector capable of expressing channel subunits in mammalian systems have been described previously [40,41]. The cDNA sequence for 〈-hENaC was cloned in frame into the pECFP-C1 (Takara Bio USA, Inc.; San Jose, CA, USA) plasmid. The resulting ECFP-tagged-〈ENaC fusion protein (CFP-〈ENaC) was used to track ENaC expression in patch clamp and CoroNa green experiments. For membrane expression experiments, the cDNA sequence for β-hENaC was cloned into the pEYFP-N1 vector (Takara Bio USA, Inc.; San Jose, CA, USA) to create “βENaC-YFP” and and γ-hENaC was cloned into a mCherry vector to create “γ-ENaC-mCherry”. EYFP and mCherry were fused to the C terminus of ENaC. Unless otherwise indicated, all three subunits were simultaneously transfected to cells with the indicated mutation to form a hetero-trimeric ENaC channel.

Site directed mutagenesis was performed by TOP Gene Technologies (St-Laurent, QC, Canada) or in the lab using the Quickchange Lightning Site-directed Mutagenesis kit (Agilent, Santa Clara, CA, USA) and standard desalted primers from Thermo Fisher (Waltham, NA, USA). The following mutants were created by substituting alanine for the indicated cationic residues: βN1 (K4A, K5A, K9A, H12A, R13A); βN2 (K39A, R40A, K47A, K48A, K49A); γN2 (R42A, R43A, R48A, R50A, R52A, R53A); γN2* (R48A, R50A, R52A, R53A); γC (R563A, R564A, K568A, K570A, K576A); and the triple mutant βN2γN2γC containing substitutions corresponding to the βN2, γN2, and γC mutants. All sequences were confirmed by standard sequencing (Psomagen, Rockville, MD, USA).

HEK 293 (CRL-1573) and CHO-K1 (CCL-61) cells were from ATCC (American Type Culture Collection, ATCC, Manassas, VA, USA). The cell permeant Na^+^ indicator, CoroNa Green AM (ThermoFisher Scientific, Waltham, MA, USA), was used to monitor intracellular [Na^+^]_i_ levels. 

### 4.2. Quantificaton of Intracellular Na^+^

The intracellular Na^+^ measurements were recorded following a protocol previously described in detail [14]. Briefly, HEK 293 cells were grown on uncoated glass bottom dishes (MatTek, No. 1.5 coverglass) and transfected with 0.25 µg of each plasmid using Fugene HD (Promega, Madison, WI, USA). Plasmids containing untagged β and γ-ENaC (wt or mutant) were transfected along with CFP-αENaC, untagged CIBN and mCh-CRY2-OCRL plasmids. Cells were kept in the dark and in the presence of 10 µM amiloride for ~48 h. Prior to imaging, cells were incubated in the dark 30 min in serum-free DMEM containing 10 µM amiloride (Sigma, Burlington, MA, USA), 5 µM CoroNa Green AM (Molecular Probes, Eugene, OR, USA) and 0.04% Pluronic F-127 (Biotium, Fremont, CA, USA), then replaced with PBS. A 60×/1.45 TIRF oil objective with 1.5× amplification on an inverted Nikon Eclipse TE2000-U fluorescence microscope was used for the CoroNa Green experiments. Imaging and blue light illumination (BLI) were performed using OBIS FP fiber pigtailed lasers (Coherent, Inc., Santa Clara, CA, USA) at 2% power. Membrane levels of mCherry-CRY2-OCRL and ENaC were quantified under TIRF settings, excitation at 561 nm and 445 nm, respectively. CoroNa Green was also examined under TIRF microscopy by excitation at 514 nm. To induce CRY2 dimerization with CIBN, we used BLI pulses (excitation at 445 nm) at 300 ms for 30 sec at a frequency of 1 Hz. Images were captured before BLI, 0 min after, 5 min after and 10 min after BLI, with an Andor iXon Ultra camera. Images were evaluated using Metamorph software (Molecular Devices, San Jose, CA, USA) and ImageJ [42]. The change in fluorescence was plotted and normalized to the starting fluorescence of each experiment. Statistical significance for each experiment was determined using GraphPad Prism 7. To compare changes in membrane expression, ordinary one-way ANOVA was performed followed by Dunnett’s multiple comparison test compared to wtENaC membrane expression on at least n = 6 cells per test. For comparison of CoroNa Green intensity changes within the same cells, significance was determined by a paired, two-tailed *t* test, and each analysis was performed on n = 11 individual cells. *, *p* < 0.0001; **, *p* < 0.005; #, *p* < 0.05; n.s., no significant difference.

### 4.3. Membrane Expression of ENaC 

The relative membrane expression levels of wt and mutant ENaC were determined using TIRF microscopy. Plasmids containing CFP-α-ENaC, βENaC-YFP (wt or mutant) and γ-ENAC-mCherry (wt or mutant) were each transfected (0.25 µg each) to HEK 293 cells using Fugene HD (Promega, Madison, WI, USA). Cells were grown on uncoated glass bottom dishes (MatTek, No. 1.5 coverglass) for ~48 h in the presence of 10 μM amiloride in DMEM + 10% FBS. Prior to imaging, the media was replaced with PBS. TIRF imaging was carried out as described above. ECFP was excited at 445 nm. EYFP was excited at 514 nm, and mCherry was excited at 561 nm. Data were collected and analyze as described above. Mean intensities were measured in ImageJ and analyzed and plotted in GraphPad Prism 7. One-way ANOVA was performed followed by Dunnett’s multiple comparison test compared to wtENaC membrane expression on at least n = 8–14 cells for each of 2 experiments. **, *p* < 0.005; *, *p* < 0.05.

### 4.4. Electrophysiology

CHO-K1 cells were grown on glass chips coated with poly-l-lysine (Sigma, St. Louis, MO, USA). Cells were transfected with 0.125 µg each α, β and γ ENaC plasmid (wt or mutant) and 0.25 µg each CIBN and mCh-CRY2-OCRL plasmid, using Lipofectamine 3000 (Invitrogen; Thermofisher, Waltham, MA, USA) as described in the manufacturer’s protocol. In brief, 60% confluent cells in a 35 mm dish were transiently transfected with the same ENaC constructs as described above, in the presence or absence of the CIBN/CRY2 constructs. Cells were kept in the dark with 10 µM amiloride. Cells were patched within 24–48 h after transfection. Whole-cell currents of ENaC were recorded under voltage clamp using the extracellular solution, (in mM) 150 NaCl, 1 CaCl_2_, 2 MgCl_2_, and 10 HEPES (pH 7.4). The pipette solution contained (in mM) 120 CsCl, 5 NaCl, 2 MgCl_2_, 5 EGTA, 10 HEPES, 2 ATP, and 0.1 GTP (pH 7.4). The current densities were recorded on an Axopatch 200B (Molecular Devices, San Jose, CA, USA) patch-clamp amplifier interfaced via a Digidata 1550B (Molecular Devices) to a PC running the pClamp 11 suite of software (Molecular Devices). All currents were filtered at 1 kHz. Cells were clamped to a 40 mV holding potential with voltage ramps (500 ms) from 60 mV down to −100 mV used to elicit current. Whole-cell capacitance, on average 8–10 pF, was compensated. Series resistance, on average 3–6 megaohms, were also compensated.

Cells were visualized with a 10× or 40× objective under BLI conditions with transmitted light. Cells expressing CFP and mCherry were found using a mercury lamp with 445 nm and 561 nm filter cubes. The starting ENaC recordings were made under 445 nm BLI-induced PIP2 depletion conditions. After approximately 3 min under BLI, the microscope and ambient room lights were switched off and the recordings were made for an additional 10 min, followed by perfusion of 10 μM amiloride. Two-way ANOVA was used to determine statistical significance of n = 6–15 cells, with the row factor defined as the mutant and the column factor defined as light vs. dark, followed by Sidak Holm’s multiple comparisons test. For the time-course experiment, two-way ANOVA was used, where row factor = time and column factor = mutant, followed by Dunnett’s multiple comparisons test.

## Data Availability

The data supporting the findings of this study are contained within the contents of this article. The datasets generated during this study will be freely provided by the corresponding author upon request.

## Supplementary Materials

The following supporting information can be downloaded at: https://www.mdpi.com/article/10.3390/ijms23073884/s1.
