# Supplementary material for: Optogenetic Control of PIP2 Interactions Shaping ENaC Activity
Source: Int J Mol Sci. 2022 Mar 31;23(7):3884. doi: 10.3390/ijms23073884 (PMC8998630; doi:10.3390/ijms23073884)
Supplement: Supplementary file 1 [file ijms-23-03884-s001.zip › ijms-1658970-supplementary.pdf]

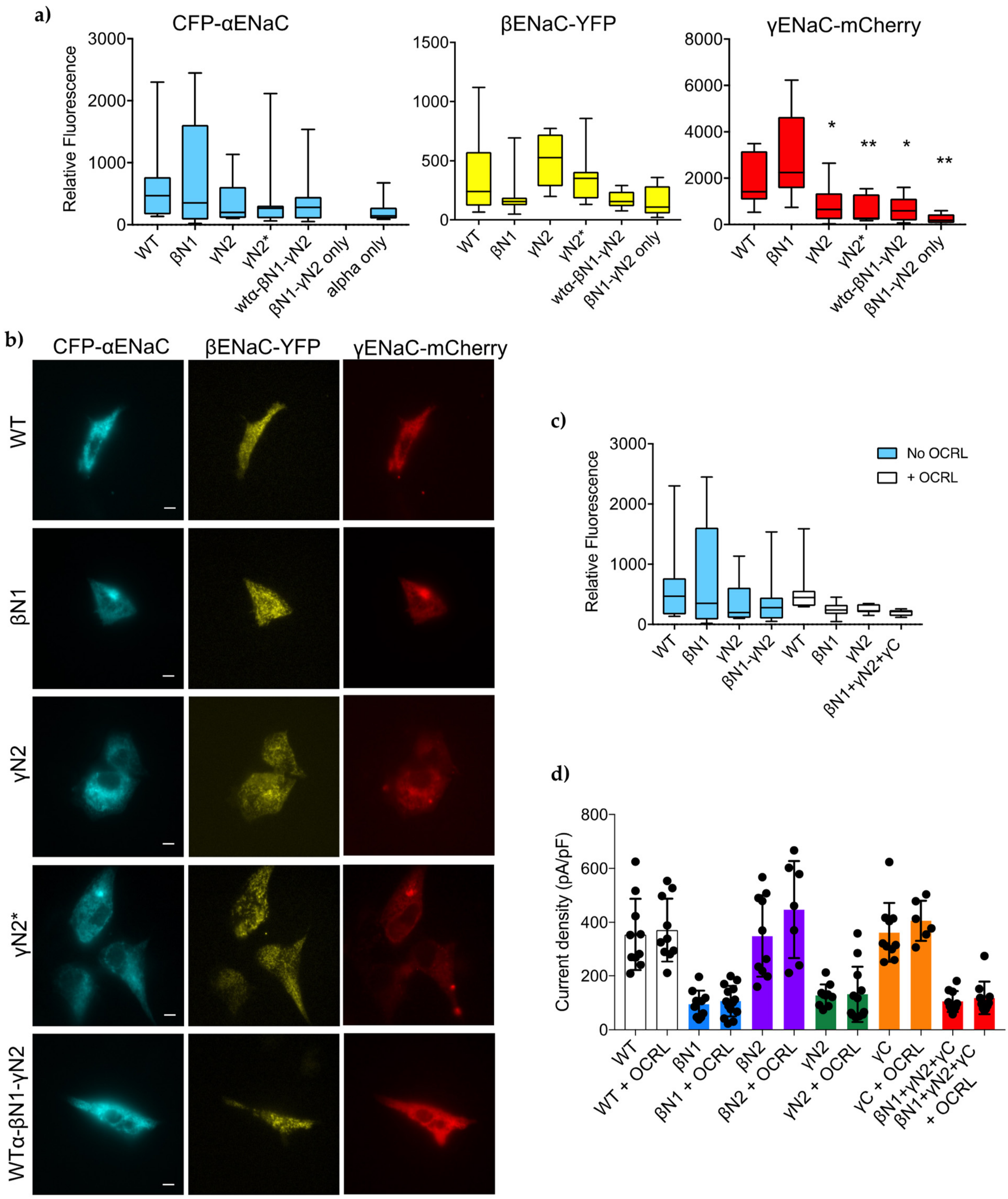

Figure S1. Membrane expression of ENaC subunits. a) Summary graphs comparing the relative fluorescence intensity of α-ENaC (left, cyan), β-ENaC (middle, yellow) and γ-ENaC (right, red) in HEK 293 cells expressing wt or the indicated mutant ENaC. **b**) Representative cells showing the relative fluorescence of CFP-α, β-YFP and γ-mCherry corresponding to the summary graphs above. White bar = 10 µm. **c)** Summary graph comparing membrane CFP expression of α-ENaC alone, or cotransfected with CIBN/mCh-CRY2-OCL in the dark. No difference was found between the groups. **d)** Summary graph comparing macroscopic current densities of wt or mutant ENaC alone or cotransfected with CIBN/mCh-CRY2-OCL. No difference was found between +/- OCRL. All data are from 6-14 cells. *, $p<0.05$, **$p<0.005$, compared to wtENaC.

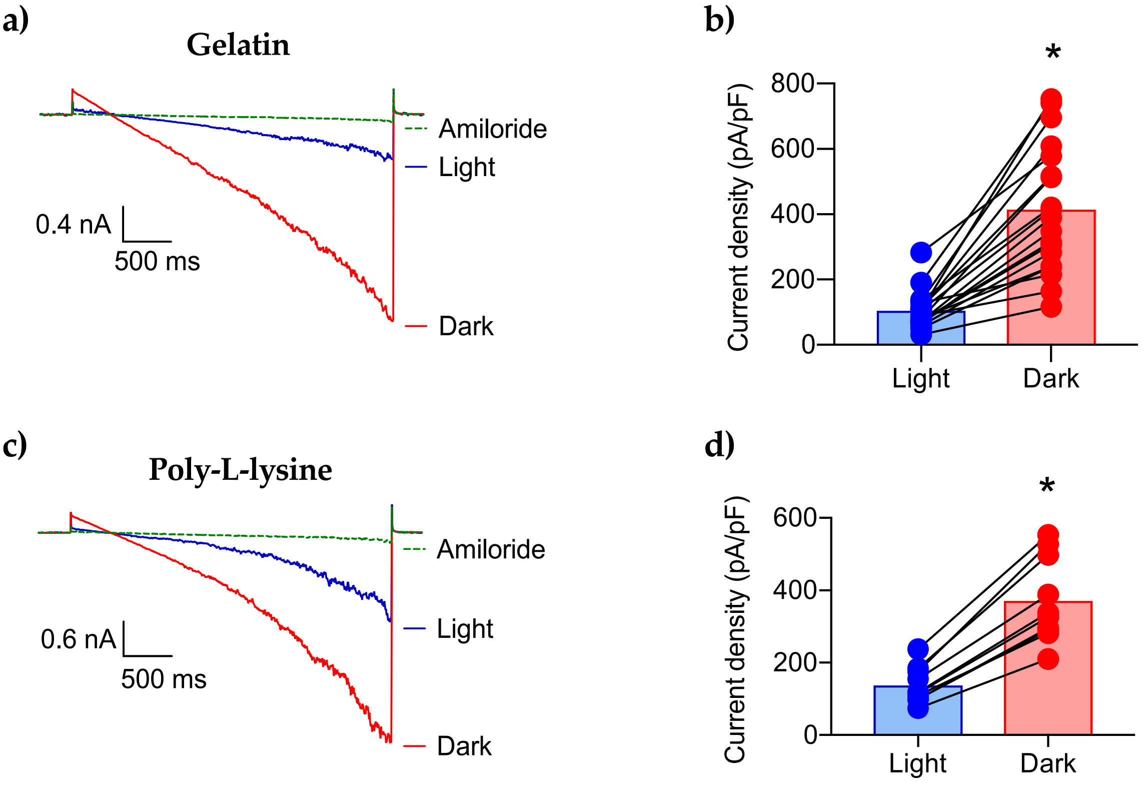

Figure S2. Comparison of PLL and gelatin on ENaC activity in response to PIP2 depletion. Representative current traces of CHO cells expressing wtENaC, **(a, c)** under BLI (1, blue trace), then dark (2, red trace), then 10 uM amiloride (3, green trace). Summary graphs of the mean current density for wtENaC **(b,d)** at -100 mV under low PIP2 levels ("Light", blue bar and blue circles) vs maximum PIP2 levels ("Dark", red bar and red circles). The wtENaC activity was compared between cells coated on gelatin, *top panel*, and cells coated on PLL *bottom panel*. *, p<0.0001.

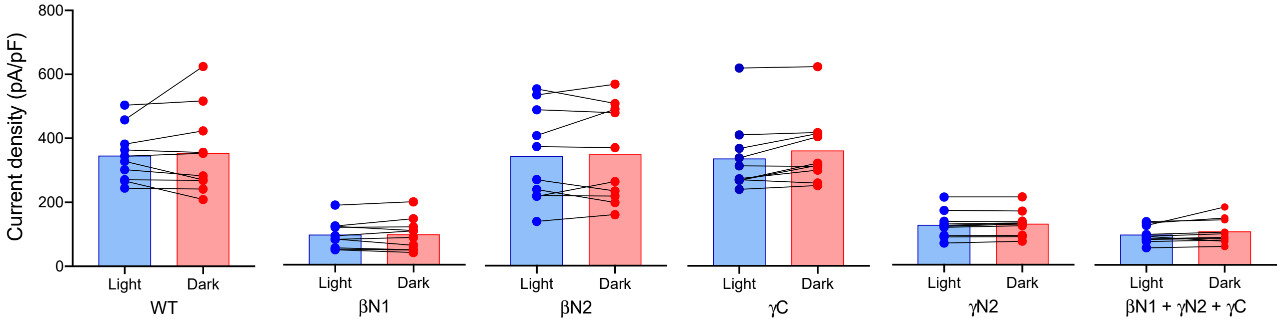

Figure S3. Current densities of wt and mutant ENaC constructs in the absence of CIBN/mCh-CRY2-OCRL. Summary graphs of the mean current densities of wt and mutant ENaC at -100 mV in CHO cells in the absence of CIBN and mCH-CRY2-OCRL. Cells were alternated with blue light (“Light”, blue bar) and dark environment (“Dark”, red bars) to show that light does not affect PIP2 levels or ENaC activity. No significant difference between the Light and Dark groups was detected.

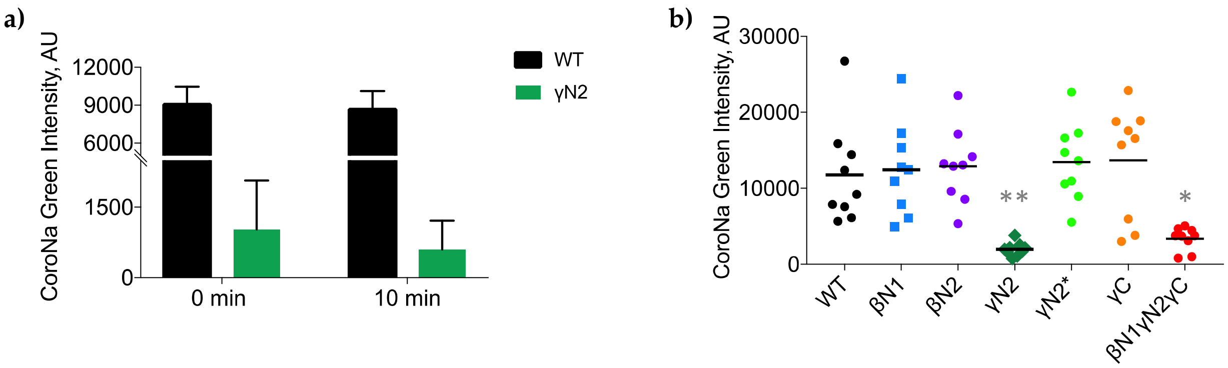

Figure S4. Comparison of CoroNa Green levels independent of PIP2 depletion. a) Summary graph of the relative fluorescence intensity of CoroNa Green AM in HEK 293 cells expressing wtENaC (black bars) or the γN2 mutant (green bars), without BLI-induced PIP2 depletion. Data are expressed as the mean of 3 cells per group ± SD. CoroNa Green levels decreased by 42 ± 5% in mutant γN2 cells over the course of 10 min, but was not significantly different. CoroNa Green levels in wtENaC were unchanged over 10 min (4.0 ± 9%). **b)** A comparison of CoroNa Green levels in HEK 293 cells expressing wt- and mutant ENaC at t=0, prior to BLI-induced PIP2 depletion. Cells expressing the γN2 mutant had significantly reduced level of CoroNa Green, indicated by one-way ANOVA, *, $p<0.05$; **,$p<0.005$, n=9 cells per group), compared to wtENaC.

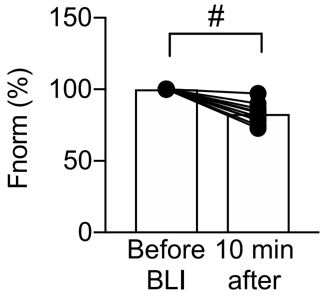

Figure S5. Effects of PIP2 depletion on the partial mutant γN2*-ENaC. Summary graph showing the normalized intensity of CoroNa Green before and 10 min after BLI, in cells containing the mutant γN2*-ENaC. This construct does not carry the R42A/R43A substitutions. CoroNa green intensity was reduced to 82.7 ± 7%. n=10 cells, #, $p<0.05$.
